# International Telepresence‐Assisted Robotic Colorectal Surgery Using the da Vinci 5 System: A Technology Paper on Cross‐Border Remote Mentoring

**DOI:** 10.1111/ases.70307

**Published:** 2026-05-06

**Authors:** Yume Minagawa, Yasumitsu Hirano, Yasuhiro Ishiyama, Sohei Akuta, Akihito Nakanishi, Yusuke Nishi, Hisashi Hayashi, Takatsugu Fujii, Hirofumi Sugita, Chikashi Hiranuma

**Affiliations:** ^1^ Department of Gastroenterological Surgery Saitama Medical University International Medical Center Saitama Japan

**Keywords:** da Vinci 5, force feedback, robotic colorectal surgery, telementoring, telepresence, visual haptics

## Abstract

**Background:**

Telepresence enables real‐time observation and remote mentoring across distances, potentially accelerating the dissemination of advanced minimally invasive techniques. The da Vinci 5 platform integrates Telepresence within the My Intuitive digital ecosystem, facilitating remote case observation, collaboration, and mentoring.

**Methods:**

This technology paper reports an international telepresence session in which an expert colorectal robotic surgeon located in Daegu, Republic of Korea, provided real‐time remote mentoring during a robot‐assisted colorectal procedure performed in Saitama, Japan. A representative clinical implementation involved a patient with rectosigmoid cancer (clinical stage T1bN0M0) and obesity (BMI 30.1 kg/m^2^). The telepresence‐mentored segment focused on the most technically demanding phase: peritoneal incision, dissection around the superior rectal artery (SRA), and vascular handling.

**Results:**

The mentored phase lasted 40 min. During this segment, the remote mentor provided step‐by‐step guidance on exposure strategy, traction direction, and plane selection. Additionally, console‐integrated objective force visualization (“Force Gauge”) was used as a shared reference to optimize retraction. The operation was completed without intraoperative complications (operative time: 214 min; estimated blood loss: 5 mL).

**Conclusion:**

These findings demonstrate the feasibility of cross‐border telepresence mentoring on the da Vinci 5 platform. Coupling remote mentoring with objective intraoperative force feedback may improve shared situational awareness and support safe decision‐making during complex robotic colorectal surgery.

## 1 Introduction

Remote collaboration in surgery spans a spectrum from tele‐observation and telementoring to full telesurgery. Since the landmark transatlantic telesurgery by Marescaux et al. in 2001 [1], technological advancements have aimed to bridge geographical distances in surgical care. While full telesurgery remains limited by regulatory hurdles and infrastructure requirements, telementoring has emerged as a practical means to extend expert guidance without travel, thereby supporting training and quality assurance, particularly in underserved or remote settings [2].

Recent studies have validated the safety and utility of robotic telementoring. Faris and colleagues reported that remote surgical consultation using a robotic platform was feasible with reliable audiovisual connectivity, positively influencing the surgical team's perception [3]. Furthermore, systematic reviews indicate that telementoring systems incorporating modern communication technologies, such as 5G and extended reality (XR), can enhance skill acquisition and procedural outcomes [2].

Robotic‐assisted surgery is well suited for remote mentoring because the operative view is digitized, allowing shared discussion with minimal disruption to the workflow. The da Vinci 5 system introduces platform‐level digital tools, including Telepresence, which is positioned as a real‐time collaboration function within the My Intuitive ecosystem. In colorectal surgery, subtle changes in traction and plane selection can significantly influence bleeding risk and tissue injury; therefore, timely expert guidance during critical phases is of high practical value.

Here, we present a case of international telepresence‐mentored robotic high anterior resection performed in Japan with remote proctoring from Korea. We focus on how remote guidance, combined with console‐integrated objective force visualization (“Force Gauge”), contributes to the surgical technique and intraoperative decision‐making during challenging vascular dissection.

## 2 Materials and Surgical Technique

A representative clinical implementation involved a 56‐year‐old male with obesity (BMI 30.1 kg/m^2^) who was diagnosed with rectosigmoid cancer staged as cT1bN0M0 based on preoperative evaluation. Robot‐assisted high anterior resection was planned using the da Vinci 5 platform. The primary surgeon at the Japanese site was a colorectal surgeon in their 9th postgraduate year. A senior colorectal robotic surgeon participated as a remote mentor from Daegu, Republic of Korea.

The remote mentoring was performed using the built‐in telepresence system integrated into the da Vinci 5 platform. The telepresence system was operated under network conditions that met the manufacturer's specified requirements for the da Vinci 5 platform. According to these specifications, telepresence requires a bandwidth of approximately 6–8 Mbps and a network latency of less than 150 ms. The end‐to‐end system latency, including processing and transmission delays, is reported to be approximately 1.45 s. In the present case, the procedure was performed under these conditions, and no apparent communication delay or instability was observed during surgery.

At the operating site, the surgical field from the robotic console was transmitted in real time via the da Vinci 5 system. At the mentor site, the remote surgeon accessed the system using a standard personal computer and monitor, without a robotic console.

Real‐time bidirectional audio communication was established through the system. In addition, the mentor utilized a visual annotation function integrated into the platform to indicate specific anatomical landmarks and optimal dissection planes directly on the shared surgical view.

The combination of verbal instructions and visual annotation enabled precise and intuitive guidance throughout the procedure.

A telepresence session was scheduled in advance. The remote mentor joined electronically, observing the live operative view and communicating in real‐time with the operating team. No remote manipulation of robotic instruments was performed, and all operative responsibility remained with the local surgeon. The mentored segment was prospectively defined as the high‐risk technical phase: peritoneal incision, mesenteric and vascular dissection around the SRA, and vascular handling.

**FIGURE 1:**
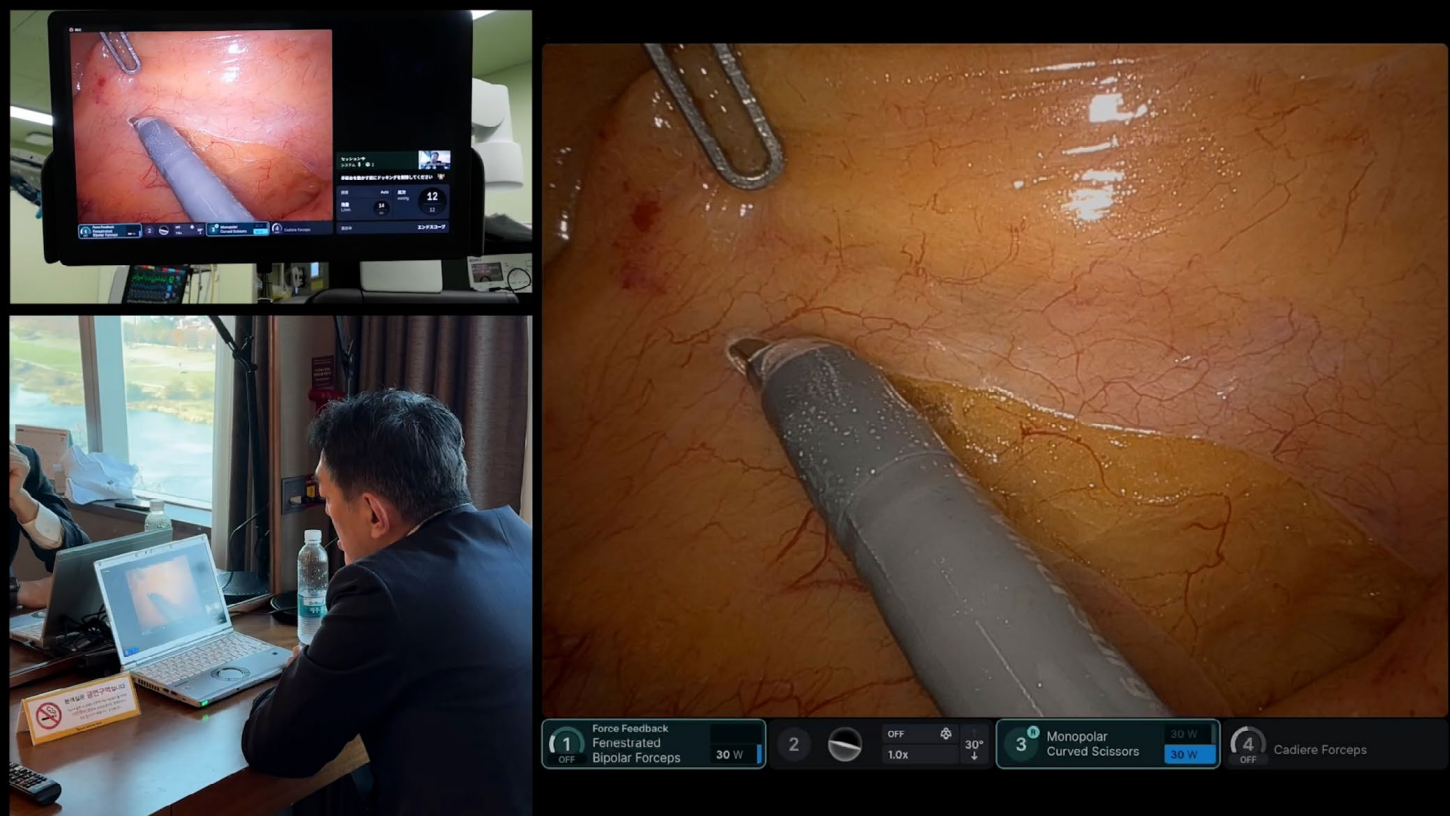
The mentor provided real‐time guidance on setting the appropriate dissection line during mesorectal incision.

**FIGURE 2:**
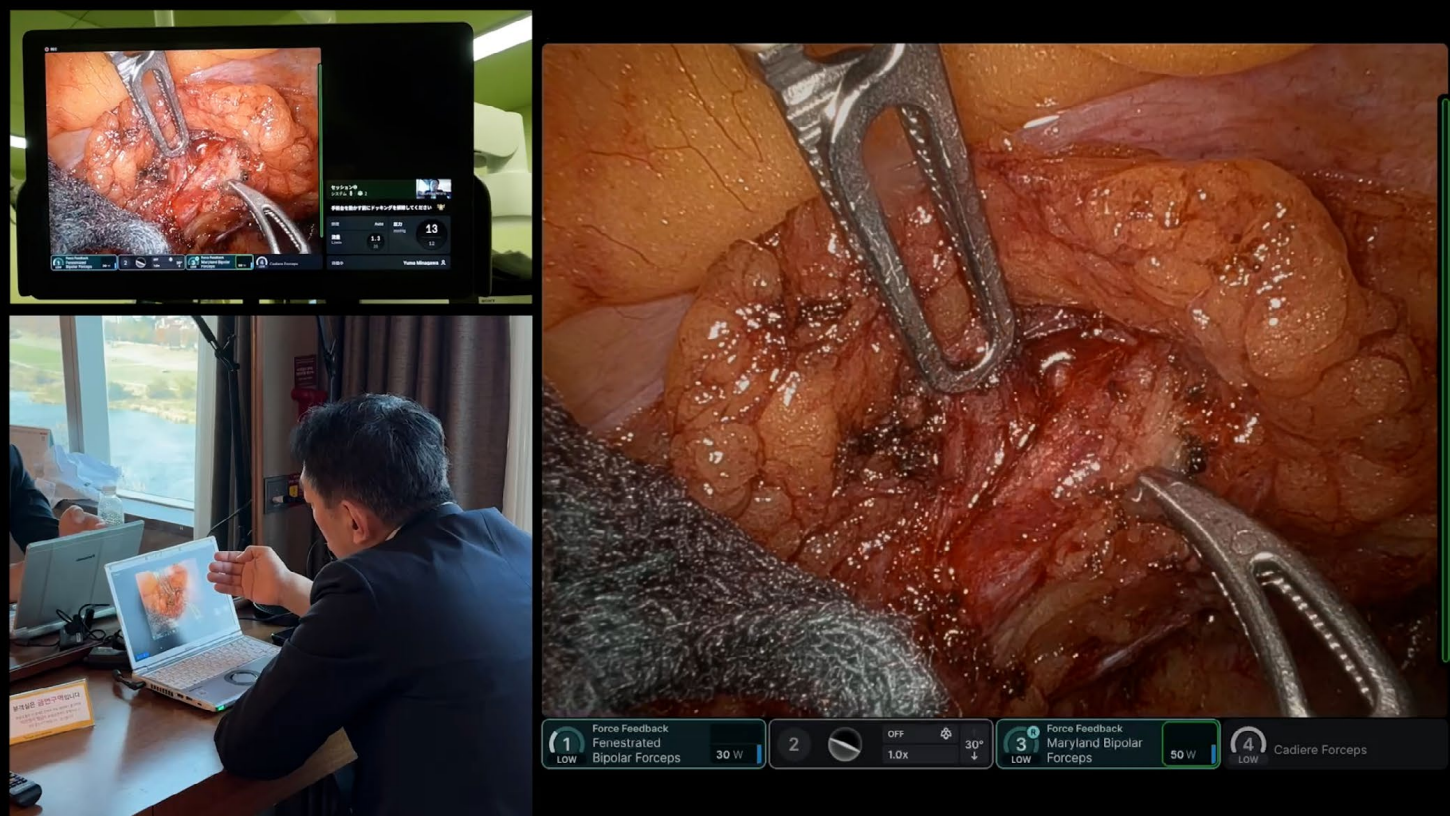
The mentor provided real‐time guidance on safe procedural steps during lymphadenectomy and vascular handling.

This mentored period lasted 40 min. During the mentored segment, the remote mentor provided continuous guidance on: (i) camera alignment and exposure strategy suitable for an obese patient; (ii) traction direction and counter‐traction to maintain a consistent dissection plane while minimizing tension on the mesentery (Figure 1); and (iii) the stepwise progression of dissection and vascular control to reduce bleeding risk (Figure 2). These steps are particularly prone to prolonged operative time due to bleeding or deviation from the correct dissection plane, especially in obese patients, and are considered phases in which inter‐operator variability is likely to occur.

Console‐integrated force visualization (“Force Gauge”) was employed as an objective reference during retraction. When elevated traction forces were observed, the mentor instructed the surgeon to adjust the grasp point and traction vector, combined with temporary relaxation and re‐application of tension to restore safer exposure. This intervention led to a change in the field development strategy, facilitating the completion of vascular dissection without bleeding or tissue injury. The total operative time was 214 min, and the estimated blood loss was 5 mL. No intraoperative complications occurred. The patient recovered uneventfully and was discharged on postoperative day 7.

The telepresence function of the da Vinci 5 system is designed based on secure communication protocols. Specifically, encrypted communication is ensured through mutual TLS authentication, allowing data transmission only in an environment where both client and server authentication are established. In addition, access control is appropriately managed through an authentication mechanism based on OAuth 2.0. Furthermore, the system operates in compliance with international security standards, including the NIST Cybersecurity Framework and ISO 27001.

The procedure was conducted in accordance with the ethical and institutional frameworks of the participating institutions, and appropriate institutional approval was obtained (IRB No. 2022‐035).

## 3 Discussion

### 3.1 Principal Findings and Novelty

This technology paper demonstrates the feasibility of cross‐border telepresence mentoring during robotic colorectal surgery using the da Vinci 5 platform. This report is notable for its international implementation (from Daegu, Republic of Korea to Saitama, Japan) and for the practical application of objective force visualization to support remote coaching. Our experience aligns with recent advancements in cross‐border telesurgical collaboration, such as the ultra‐long‐distance telesurgery reported between Asia and the Middle East [4], confirming that international remote collaboration is becoming increasingly viable.

### 3.2 Telepresence in the Context of Telementoring Literature

Telementoring is defined as real‐time guidance provided by a remotely located expert. Reviews have highlighted its potential to reduce travel‐related barriers and expand access to subspecialty guidance [2]. Recent feasibility studies confirm that reliable connectivity and clear audiovisual delivery are achievable, reinforcing that the critical safety condition is the continuous presence of a credentialed surgeon at the patient's side [3]. Telepresence on the da Vinci 5 leverages the My Intuitive ecosystem, adhering to third‐party certifications for privacy and security. This integration may lower practical barriers compared with ad hoc solutions by aligning with standardized workflows, addressing the need for communication redundancy and system reliability emphasized in recent literature [5].

### 3.3 Why Objective Force Visualization Matters for Remote Mentoring

A practical limitation of remote proctoring is the “loss of haptic context.” In robotic surgery, surgeons often rely on visual cues (“visual haptics”) to infer applied force. These cues may be less reliable in patients with obesity, where thickened mesentery necessitates higher traction. Objective force visualization bridges this gap. Reiley et al. demonstrated that visual force feedback significantly reduces suture breakage rates and peak applied forces, particularly among novice robotic surgeons [6]. Similarly, basic research suggests that force feedback reduces tissue trauma and errors [7]. In our implementation, the Force Gauge served as a common quantitative language. It allowed the remote mentor to recommend concrete modifications based on objective data rather than subjective visual estimation, improving shared situational awareness during time‐sensitive phases. Based on the force values, the mentor provided practical guidance such as adjusting the grasping point to distribute load, modifying the traction direction to improve exposure without increasing force, and temporarily releasing tension to re‐identify the dissection plane before continuing. An on‐site backup surgeon assisted in translating these verbal instructions into precise maneuvers.

### 3.4 Implementation Considerations

International telepresence introduces considerations regarding data governance and credentialing. Based on prior literature [3, 5], we recommend: (i) a pre‐case briefing with shared terminology; (ii) standardized escalation rules; and (iii) explicit patient consent describing remote participation. Future developments should also consider the role of 5G and AI‐driven assessment to further enhance the fidelity of remote mentorship, as suggested in recent systematic reviews [2].

### 3.5 Limitations

This report represents a single case experience. Objective network metrics (latency, jitter) were not recorded for research purposes, although no perceptible delay hindered the mentorship. Further prospective evaluations are warranted to define best practices and measure the clinical impact of telepresence‐enabled mentoring. In this study, detailed measurements of network parameters such as latency and jitter were not recorded, which represents a limitation. Future studies including quantitative evaluation of network performance are warranted.

## 4 Conclusion

Cross‐border telepresence‐based remote mentoring using the da Vinci 5 platform was feasible during robot‐assisted colorectal surgery. Telepresence contributed to intraoperative decision‐making, and objective force visualization (“Force Gauge”) served as a vital shared reference to optimize retraction. This technology holds promise for globalizing surgical education and expertise.

## Funding

The authors have nothing to report.

## Conflicts of Interest

Yasumitsu Hirano has received honoraria from Intuitive Surgical Japan for invited lectures. The remaining authors declare no conflicts of interest.

## Data Availability

The data that support the findings of this study are available from the corresponding author upon reasonable request.
